# Keratin-Integrated Latex–Hydrogel Coatings: Biopolymer Design for Functional Agrotextile Materials

**DOI:** 10.3390/molecules31091544

**Published:** 2026-05-06

**Authors:** Mirosława Prochoń, Szymon Szczepanik, Oleksandra Dzeikala, Robert Adamski

**Affiliations:** 1 Institute of Polymer and Dye Technology, Lodz University of Technology, Stefanowskiego 16, 90-537 Lodz, Poland; 2 Faculty of Process and Environmental Engineering, Lodz University of Technology, Wólczanska 213, 90-924 Lodz, Poland

**Keywords:** industrial by-products, functional agrotextiles, biodegradable coatings, keratin hydrolysates, gelatin-based fertilizer, soil moisture retention, sustainable agriculture

## Abstract

This work introduces a circular biopolymer-based strategy for valorizing keratin-rich industrial residues through the fabrication of biodegradable cotton agrotextiles functionalized with latex–hydrogel coatings. Keratin hydrolysates and gelatin-derived biofertilizer capsules were incorporated into polymer–hydrogel matrices and applied onto cotton substrates to enhance soil moisture regulation and controlled nutrient release. The composite coatings were characterized in terms of water absorption capacity, mechanical performance, biodegradation profiles, and their impact on plant growth using *Phaseolus vulgaris* as a model species. Hydrogel-rich formulations (LH20 and LH40Z) provided the most favorable balance of tensile strength and controlled degradation while significantly increasing soil moisture availability and overall plant biomass compared with uncoated controls. The gelatin–keratin microcapsules enabled sustained nutrient release and induced a slight increase in soil pH, further supporting plant development. These findings demonstrate the dual functionality of the developed latex–hydrogel coatings as water-management and nutrient-delivery systems and highlight the potential of keratin biowaste upcycling toward high-value, biodegradable agricultural materials aligned with circular economy principles.

## 1. Introduction

The increasing demand for sustainable agricultural practices and the need to mitigate environmental degradation have stimulated interest in biodegradable alternatives to conventional agrochemicals and synthetic auxiliaries. Agricultural production has traditionally relied on synthetic agrotextiles, mainly based on polyolefins such as polypropylene and polyethylene, to regulate soil temperature, suppress weeds, and reduce water evaporation. However, these polymers show strong resistance to degradation in natural environments, persisting for decades and contributing to microplastic accumulation and long-term ecological impacts [1,2]. Their presence disrupts soil microbial communities, interferes with nutrient cycling, and negatively affects plant development [3,4].

In response, research has focused on agrotextiles derived from renewable and biodegradable polymers. Polysaccharides (e.g., cellulose, starch, chitosan) and proteins (e.g., keratin, collagen, gelatin) are sourced from natural feedstocks and undergo enzymatic or microbial degradation to benign end-products such as carbon dioxide, water, and biomass [5,6]. Their structural versatility and tunable functional properties—achieved through blending or chemical modification—enable tailored mechanical and environmental performance [7,8]. Integrating such materials into crop management systems reduces dependency on persistent plastics, supports soil regeneration, and aligns with sustainability directives.

Among protein-based biopolymers, keratin has gained attention due to its mechanical robustness, biodegradability, and nitrogen-rich composition [9]. Poultry feathers and bovine hair, produced as abundant industrial by-products, provide cost-effective feedstocks for keratin recovery [10,11]. Hydrolyzed keratin, obtained via enzymatic or alkaline processes, is rich in bioavailable amino acids that improve soil fertility and stimulate plant growth Studies have shown that keratin hydrolysates enhance root development, nitrogen availability, and biomass accumulation, indicating their potential as biofertilizers in sustainable agriculture [12].

Gelatin, a collagen-derived biopolymer, is increasingly investigated for use as a biodegradable encapsulation material [13,14]. Owing to its gel-forming ability, biocompatibility, and low toxicity, gelatin enables the design of controlled-release systems for nutrients. Encapsulation of keratin hydrolysates in gelatin matrices protects the active compounds and allows gradual nutrient release into the rhizosphere [15,16]. This approach enhances nutrient uptake efficiency, reduces leaching, and minimizes environmental impact. The combination of keratin and gelatin thus provides a synergistic, biodegradable platform for sustainable nutrient management.

Water retention is another central challenge in agriculture, particularly under arid and semi-arid conditions [17]. Hydrogels, especially those based on poly(vinyl alcohol) (PVA), have been developed to address this issue. Crosslinked PVA networks absorb and retain large amounts of water, releasing it gradually according to plant demand. These hydrogels are biodegradable, non-toxic, and mechanically tunable through crosslinking density and copolymerization [18,19]. Their application as soil additives or textile coatings stabilizes hydration conditions, decreases irrigation frequency, and enhances resilience to drought stress. The performance of hydrogel-based systems can be further improved by blending with biodegradable latex binders such as carboxylated styrene–butadiene rubber (XSBR). This copolymer enhances adhesion, uniformity, and mechanical durability of coatings on textile substrates, while bio-based and biodegradable rubber analogues are being developed to improve environmental profiles [20]. Latex–hydrogel composites thus represent multifunctional agrotextiles capable of maintaining soil moisture, supporting nutrient delivery, and ensuring structural stability.

The integration of hydrogel matrices with gelatin-encapsulated keratin fertilizers introduces dual functionality by simultaneously addressing water retention and nutrient efficiency. Such systems replicate natural nutrient and hydration cycles, reduce environmental runoff, and improve resource-use efficiency, thereby supporting sustainable crop production. The present study contributes to this field by designing and evaluating cotton-based agrotextiles coated with latex–hydrogel composites and functionalized with gelatin–keratin capsules. Their structural, environmental, and agronomic performance was investigated using *Phaseolus vulgaris* as a model species, with the aim of advancing bio-based agrotextiles for sustainable agriculture.

## 2. Results

### 2.1. Biological Degradation

Biodegradation of the agrotextile composites was evaluated under composting conditions for 30 days, with assessments focused on macroscopic integrity, surface appearance, and colorimetric changes (ΔE*ab). The uncoated cotton reference exhibited complete disintegration by day 30, precluding calculation of the aging coefficient and confirming high soil biological activity toward cellulose substrates. In contrast, latex–hydrogel–coated fabrics underwent partial degradation only. Typical features included surface matting, localized brown discolorations, and occasional blistering; the textile architecture remained discernible at study end.

Gelatin capsules containing keratin-based biocomponents showed a reproducible time course of transformation following burial in soil. Capsules swelled within 6 h without rupture and retained gross integrity through 24 h. Onset of shell decomposition was observed at 48 h, coincident with release of the encapsulated material. Capsules filled with feather keratin or its hydrolysate produced transient pale residues on the soil surface; all residues were visually absent by day 5, indicating dispersion into the matrix.

**Figure 1:**
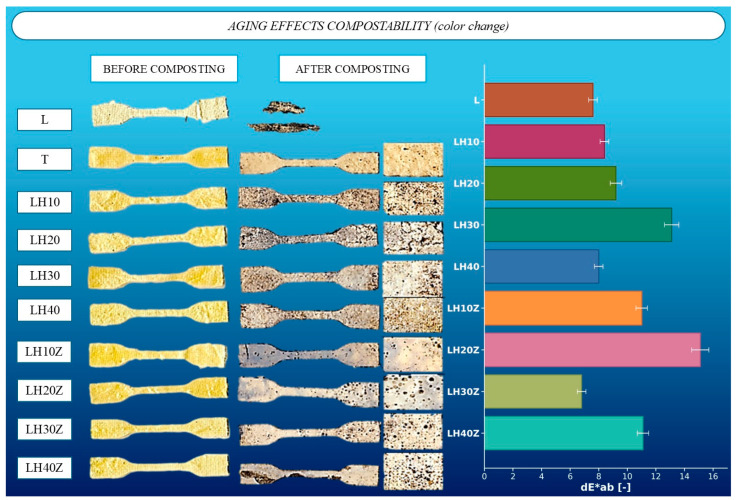
Effect of biological aging on the compostability of latex–hydrogel agrotextile composites, including visual changes before and after composting and color difference (ΔE*ab) analysis of selected samples after 30 days of soil exposure.

Colorimetry corroborated the visual observations (Figure 1). The uncoated cotton control could not be analyzed for an aging coefficient due to loss of material. Among coated samples, hydrogel-containing but gelatin-free formulations (LH20, LH30, LH40) exhibited the smallest color differences and retained the highest aging coefficients over 30 days, consistent with comparatively slower biological degradation. The LH10 formulation showed the lowest stability within this set, with an aging coefficient substantially reduced relative to LH20–LH40. Incorporation of gelatin into the coating matrix was associated with lower durability under composting; however, the LH40Z sample (40 wt% hydrogel with gelatin) maintained aging coefficients comparable to LH20 and LH30, indicating preserved resistance within that composition window [1,5,6,8].

### 2.2. Thermo-Oxidative Aging and Mechanical Stability of Latex–Hydrogel Composites

**Figure 2:**
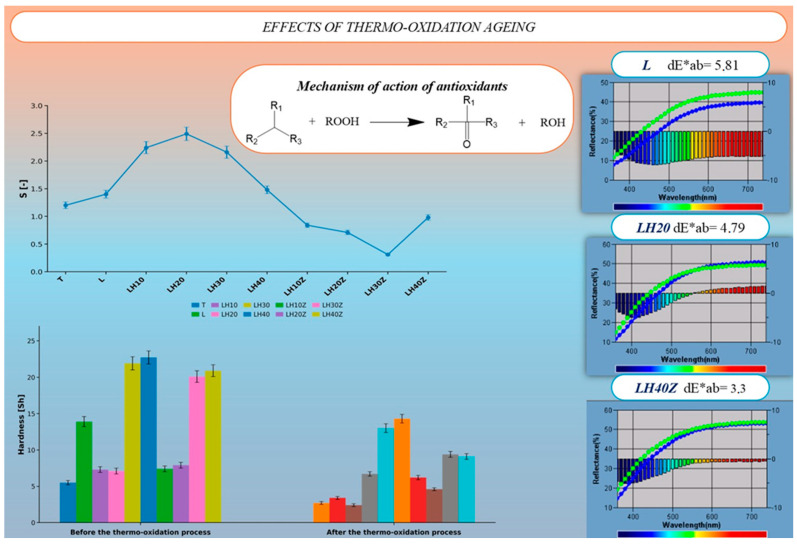
Comprehensive assessment of thermo-oxidative aging in latex–hydrogel agrotextile composites: Aging coefficient (S) across all formulations and schematic representation of the antioxidant mechanism (radical neutralization by hydrogel/latex matrix); Shore A hardness values before and after thermal exposure; Reflectance spectra and total color difference (ΔE*ab) for representative samples (L, LH20, LH40Z).

Thermo-oxidative aging of the latex–hydrogel composites was evaluated through the aging coefficient (S), Shore A surface hardness, and total color change (ΔE*ab). Figure 2 summarizes the obtained data and illustrates the antioxidative mechanism whereby hydroperoxides (ROOH) are neutralized by electron-donating moieties within the composite matrix [21].

Thermo-oxidative aging produced clear differences among the tested formulations. The aging coefficient (S) depended strongly on composition. In the gelatin-free series, S showed a non-linear trend with increasing hydrogel content, reaching the highest value for LH20 and then decreasing in the formulations with higher hydrogel loading. In the gelatin-containing series, the S values were lower overall, and the lowest value was observed for LH30Z. These results show that both hydrogel content and gelatin addition influenced the thermo-oxidative behavior of the composites.

A similar pattern was observed for surface hardness. Before aging, the highest hardness values were recorded for the formulations containing 30–40 wt% hydrogel, whereas the uncoated textile, the latex-only sample, and the low-hydrogel formulations were distinctly softer. After thermo-oxidative aging, all samples showed a decrease in hardness, indicating softening of the material under thermal exposure. Despite this decrease, the LH30 and LH40 formulations retained the highest hardness values after aging, showing better mechanical stability than the other samples.

Colorimetric analysis also revealed visible differences after thermal treatment. The latex-only sample showed the largest total color change, while the hydrogel-containing composites exhibited lower ΔE*ab values. Among the samples presented in Figure 2, the smallest color change was observed for LH40Z. The results indicate that the thermo-oxidative response of the composites was governed by coating composition, with hydrogel content having the strongest effect and gelatin modifying this behavior depending on the formulation.

### 2.3. Mechanical Properties of Latex–Hydrogel Composites

**Figure 3:**
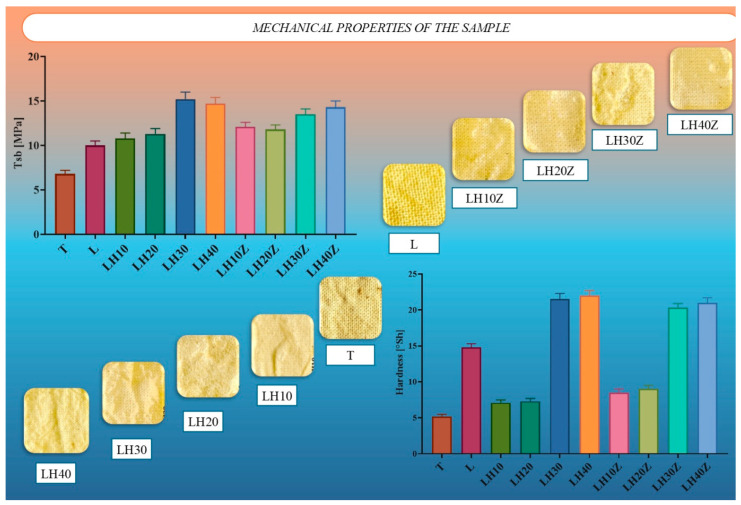
Mechanical properties of latex–hydrogel composites: (top left) Tensile strength (Tsb) of uncoated (T), latex-coated (L), and hydrogel- or gelatin-modified formulations; (top right and bottom) Microstructural appearance and Shore A hardness values of the surface, showing increasing stiffness with hydrogel content.

The mechanical performance of the coated agrotextiles was characterized by tensile strength (Tsb) and Shore A surface hardness (Figure 3).

The mechanical properties of the latex–hydrogel composites depended strongly on formulation. The uncoated cotton showed the lowest tensile strength, while latex coating alone improved this parameter. A further increase was observed after hydrogel addition, with the highest tensile strength recorded for the formulations containing 30–40 wt% hydrogel, particularly LH30. The gelatin-containing series showed a similar pattern, especially at higher hydrogel contents.

Surface hardness followed the same general trend. The uncoated fabric was the softest sample, whereas the latex-coated material was distinctly harder. Formulations with 10–20 wt% hydrogel remained relatively soft, while those containing 30–40 wt% hydrogel showed a marked increase in hardness. The highest values were observed for LH40 and the corresponding high-hydrogel gelatin-containing samples. These results show that the best mechanical performance was obtained for the high-hydrogel formulations [6,8].

### 2.4. Structural Analysis and Compostability of Latex–Hydrogel Composites: FTIR Insights

**Figure 4:**
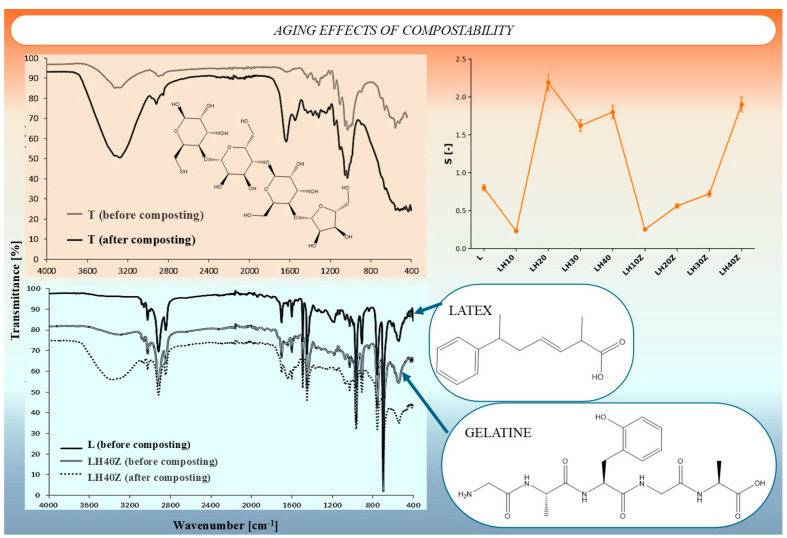
Structural analysis of compostability effects using FTIR spectroscopy: (Top left) changes in spectral profile of uncoated cotton (T) before and after composting; (Bottom) spectra of latex-only sample (L) and hydrogel–gelatin composite (LH40Z), showing degradation-induced transformations; (Top right) aging coefficient (S) across composite formulations, correlated with chemical changes; (Inset) molecular structures of latex and gelatin components indicating reactive moieties prone to hydrolysis or oxidation.

Fourier transform infrared spectroscopy (FTIR) was used to evaluate the structural changes in the latex–hydrogel agrotextiles before and after composting. Representative spectra for the uncoated cotton control (T), the latex-coated sample (L), and the gelatin-containing formulation (LH40Z) are shown in Figure 4 together with the corresponding aging coefficient (S) values.

In sample T, composting caused a clear increase in the broad absorption band in the 3600–3000 cm^−1^ region and distinct changes in the fingerprint region, especially between 1200 and 900 cm^−1^, indicating degradation of the cellulose-rich substrate during biological aging. In contrast, the latex-coated sample L showed smaller changes in the hydroxyl region, which is consistent with the more hydrophobic character of the latex layer. The LH40Z sample showed broad absorption in the 3600–3000 cm^−1^ region before composting and lower transmittance after composting in the 1650–1550 cm^−1^ and 1100–800 cm^−1^ ranges, which indicates structural changes in the hydrogel–gelatin-containing coating during biological aging [22,23].

The aging coefficient confirmed that the composting response depended strongly on formulation. In the gelatin-free series, the biological aging response showed a non-linear trend, with the highest values observed for the intermediate hydrogel formulations. In the gelatin-containing series, the values were generally lower at low and intermediate hydrogel contents, while LH40Z showed a markedly higher value than the remaining gelatin-containing samples. These results show that both hydrogel content and gelatin addition influenced the composting behavior of the coated textiles.

### 2.5. Equilibrium Swelling

The equilibrium swelling behavior and corresponding relative crosslinking coefficient (Table 1) were analyzed to evaluate how hydrogel and gelatin content affect the hydrophilic properties of the latex–hydrogel agrotextile composites. Water served as the swelling medium under ambient conditions.

The latex-only sample (L) showed the lowest equilibrium swelling ratio and the highest relative crosslinking coefficient, which indicates a compact network structure with limited water uptake. After hydrogel incorporation, the swelling capacity increased markedly in the gelatin-free series. The highest swelling was observed for LH20, which also showed the lowest relative crosslinking coefficient. This confirms that hydrogel addition increased the hydrophilic character of the coatings and reduced network density.

The addition of gelatin affected this behavior mainly at low hydrogel contents. In the LH10Z and LH20Z formulations, swelling decreased by about 43% and 40%, respectively, compared with the corresponding gelatin-free samples. At the same time, the relative crosslinking coefficient increased, showing that gelatin promoted a denser structure in these coatings and thereby restricted water diffusion.

At higher hydrogel contents, the effect of gelatin became much smaller. The swelling ratios and relative crosslinking values of LH30/LH30Z and LH40/LH40Z remained very similar, indicating that the hydrogel phase became the main factor controlling water uptake in these formulations. The results show that hydrogel increased swelling, while gelatin reduced this effect mainly in the low-hydrogel systems [8].

### 2.6. Solubility in Water

**Figure 5:**
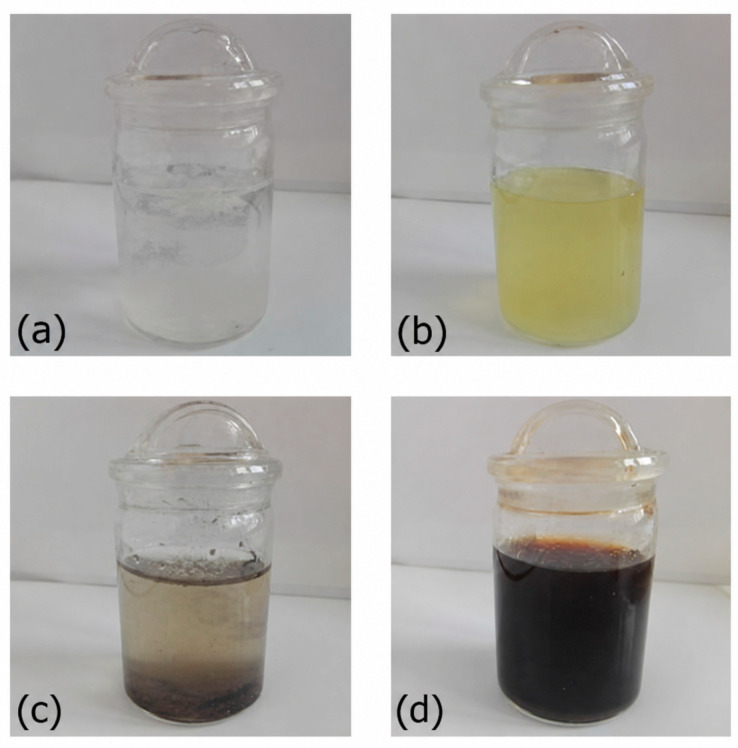
Water solubility of gelatin capsules containing biocomponents: (**a**) feather keratin, (**b**) feather keratin hydrolysate, (**c**) bovine hair keratin, (**d**) bovine hair keratin hydrolysate.

The dissolution behavior of gelatin capsules containing different keratin-based biocomponents was assessed in distilled water by visual observation of capsule swelling, shell rupture, dispersion of the released material, and sediment formation (Figure 5). The results showed that the dissolution pattern depended strongly on the form of keratin used as the capsule filling.

Capsules containing keratin hydrolysates derived from feather and bovine hair began to disintegrate within the first 2 h of immersion. The capsule shells gradually softened and ruptured, releasing the hydrolyzed material into the aqueous medium. After release, the contents formed relatively uniform, translucent dispersions, which is consistent with the higher water compatibility of the hydrolyzed keratin fractions.

In contrast, capsules filled with native keratin powders showed slower and less complete dissolution. During the first 2 h, these capsules showed visible swelling, but most of them still retained their general structure. Rupture was observed after about 2.5 h, followed by the formation of turbid suspensions. In these samples, visible sediment remained at the bottom of the vessel, indicating incomplete dissolution and limited dispersibility of the native keratin material.

Empty gelatin capsules, used as controls, initially floated on the water surface because of their low density. After gentle mechanical agitation, they dissolved completely within about 40 min, producing a clear and colorless solution without visible residue. These observations show that the physical form of keratin had a clear effect on capsule disintegration and on the appearance of the released material in water. Hydrolyzed keratin produced more uniform dispersions, whereas native keratin led to heterogeneous suspensions with visible sediment.

### 2.7. pH Measurement After Capsule Dissolution

The pH values of the aqueous solutions obtained after dissolution of gelatin capsules were measured for the empty capsules and for capsules filled with keratin-based materials (Table 2).

The pH measurements showed clear differences between capsules filled with native keratin and those containing keratin hydrolysates. The empty gelatin capsule produced a nearly neutral solution, close to that of water, and the capsules filled with native feather keratin or native bovine hair keratin also remained in the near-neutral range. This indicates that neither the capsule shell nor the native keratin fillers markedly changed the reaction of the aqueous medium.

In contrast, the capsules containing feather keratin hydrolysate and bovine hair keratin hydrolysate produced mildly alkaline solutions. Their pH values were higher than those of the empty capsule and the native-keratin systems, which is consistent with the chemical nature of the hydrolyzed materials. The pH results clearly distinguish the native keratin samples, which remained close to neutral, from the hydrolysate-filled capsules, which shifted the medium toward mild alkalinity after dissolution [24].

### 2.8. Thermal Stability of Gelatin Capsules

The thermal stability of the empty gelatin capsules was evaluated using 10 samples at each storage temperature in order to assess their physical integrity during handling and storage. The capsules were stored at 30 °C and −4 °C for 5 days, and their appearance was inspected daily. In both cases, all capsules remained intact, and no visible deformation, cracking, or adhesion was observed. Although the melting range of a 10% gelatin gel is reported to be 29 ± 2.5 °C, the capsules maintained their structure at 30 °C, indicating adequate stability under these conditions [22]. 

Additional tests were carried out at −20 °C and 50 °C. After storage at −20 °C, capsules retained their original appearance, with no visible brittleness, delamination, or discoloration. At 50 °C only slight yellowing of the capsules was observed. These observations show that the gelatin capsule shells preserved their physical integrity over the tested temperature range from −20 °C to 50 °C.

### 2.9. Plant Growth Assessment Using Keratin-Based Fertilizers and Latex–Hydrogel Agrotextiles

**Figure 6:**
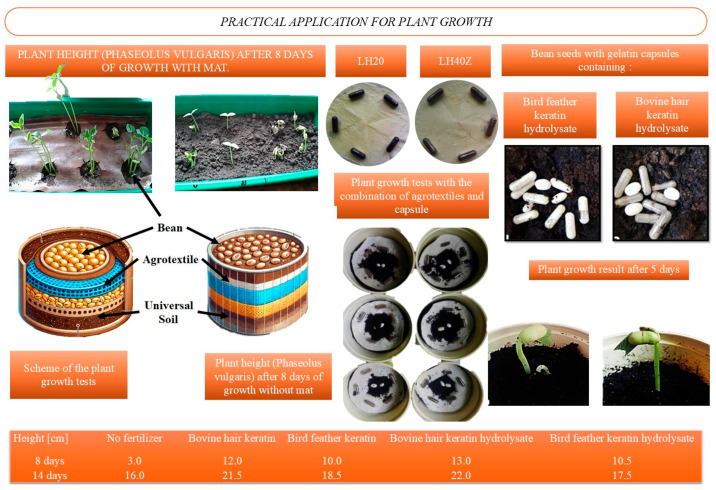
Evaluation of early *Phaseolus vulgaris* growth using latex–hydrogel agrotextiles and keratin-based gelatin capsules: (**Top left**) comparison of plant height with and without agrotextile mat after 8 days; (**Top right**) LH20 and LH40Z samples used for combined capsule–agrotextile growth tests; (**Middle**) test scheme showing layered configuration of soil, agrotextile, and seed placement; (**Bottom right**) plant development after 5 days using keratin hydrolysate capsules; (**Bottom row**) quantitative results of plant height after 8 and 14 days using different fertilizers.

The early growth performance of *Phaseolus vulgaris* (common bean) was evaluated using latex–hydrogel agrotextile mats combined with gelatin capsules containing keratin-derived fertilizers. The experimental setup (Figure 6) included control plants grown directly in soil and test groups grown on agrotextiles LH20 and LH40Z, representing latex coatings with 20 wt% and 40 wt% hydrogel, respectively.

The plant-growth test showed clear differences between the unfertilized system and the capsule-fertilized treatments. All keratin-based capsule formulations improved early growth relative to the no-fertilizer group, but the magnitude of the effect depended on the type and form of keratin used. The strongest response was observed for the capsules containing bovine hair keratin hydrolysate, which gave the highest plant height at both measurement times.

Capsules filled with bovine hair keratin also promoted strong growth, whereas both feather-derived materials produced lower values. The results indicate that keratin hydrolysates were more effective than native keratin under the tested conditions, and that the bovine hair-derived hydrolysate had the most favorable effect on early shoot development [25].

For clarity, the obtained results have been compiled into a comparative summary table that is provided in the Supplementary Materials (Table S1).

## 3. Discussion

The obtained results show that the performance of the developed agrotextiles was governed mainly by the balance between the latex matrix, the hydrogel phase, and gelatin addition. In general, hydrogel incorporation increased the hydrophilic character of the coatings, improved water uptake, and contributed to better mechanical performance, while the latex phase limited excessive swelling and provided structural support. Gelatin acted as an additional modifier of the network structure, especially at lower hydrogel contents, where it reduced swelling and changed the degradation behavior. These observations indicate that the properties of the coatings can be tuned through formulation design, depending on whether greater durability or faster post-use degradation is required [1,5,6,8,23].

This formulation-dependent behavior was also reflected in the aging studies. Both biological and thermo-oxidative aging showed that the response of the composites was not controlled by a single component, but by the overall organization of the coating network. The FTIR results support this interpretation, as they indicate that both the cellulose substrate and the coating components participated in the degradation process. At the same time, the mechanical data show that hydrogel-rich coatings retained better structural integrity than the low-hydrogel systems, which suggests that the hydrogel phase contributed not only to water retention but also to mechanical stabilization of the composite. Taken together, these findings show that the latex–hydrogel system acted as a multifunctional coating in which barrier properties, swelling behavior, and degradation resistance remained closely interrelated [21,22,23].

The behavior of the fertilizer capsules was consistent with this material design. Capsules filled with keratin hydrolysates dissolved more readily and produced more uniform dispersions than capsules containing native keratin, while also shifting the medium toward mild alkalinity. This suggests that hydrolyzed keratin is more suitable for rapid nutrient availability. The plant-growth results support this conclusion, as the hydrolysate-based systems gave a more favorable response than the native keratin formulations. In this way, the present study suggests that hydrogel-coated agrotextiles and keratin-based capsules may act complementarily, with the coating supporting moisture retention and the capsules providing a source of nutrients after release [24,25].

At the same time, these conclusions should be treated as application-oriented guidelines rather than universal rules. The present work was focused on the development and initial evaluation of biodegradable agrotextiles based on keratin-rich waste, and factors such as soil type, pH, microbial activity, and moisture regime were not examined separately. In addition, broader agricultural use of keratin hydrolysates will require better standardization of their composition. For this reason, the present study should be regarded as a proof of concept showing that waste-derived keratin can be incorporated into biodegradable agrotextile systems with adjustable water uptake, durability, and fertilizer-release behavior, while further optimization should be addressed in future studies under more specific soil and field conditions [26].

## 4. Materials and Methods

### 4.1. Materials

The following materials and reagents were used in this study: bovine hair keratin obtained from Kalskór S.A. (Kaliskie Zakłady Garbarskie, Kalisz, Poland); avian feather keratin sourced from Cedrob S.A. (Poultry Processing Plant, Niepołomice, Poland); the proteolytic enzyme Nue 12 MG supplied by Novozymes A/S (Bagsværd, Denmark); analytical grade sodium hydroxide from Eurochem BGD Sp. z o.o. (Tarnów, Poland); 96% sulfuric acid from P.P.H. STANLAB Sp. J. (Lublin, Poland); poly(vinyl alcohol) with a molecular weight of 72,000 from POCH S.A. (Gliwice, Poland); sodium tetraborate (borax) from Chem Point Sp. z o.o. (Kraków, Poland); carboxylated styrene-butadiene latex (LBSK, grade 5545, with a solid content of 50 ± 2% and a maximum viscosity of 100 mPa·s) from Synthos S.A. (Oświęcim, Poland); gelatin provided by Gellwe, FoodCare Sp. z o.o. (Zabierzów, Poland); cotton fabric with a grammage of 125 g/m^2^ from P.P.H.U. STILTEX Sp.J. (Pabianice, Poland); gelatin capsules (size 1) from NANGA (Złotów, Poland); common bean seeds (*Phaseolus vulgaris*, cv. Laponia) from PlantiCo Hodowla i Nasiennictwo Ogrodnicze Zielonki Sp. z o.o. (Stare Babice, Poland); universal potting soil with a pH of 5.5–6.0 from Pokon (Poznań, Poland).

### 4.2. Hydrolysis of Keratin Derived from Bovine Hair and Poultry Feathers

Keratin-rich waste materials, namely ground bovine hair and poultry feathers, were subjected to a two-stage hydrolysis process to obtain bioavailable keratin hydrolysates. The initial step involved alkaline hydrolysis using 0.25 M NaOH at 85 °C for 2.5 h, enabling the disruption of disulfide cross-links and the initial depolymerization of keratin. Subsequently, enzymatic hydrolysis was carried out by introducing a proteolytic enzyme into the reaction mixture [27], followed by incubation at 50 °C for 3 h to further cleave the polypeptide chains into low-molecular-weight fractions with enhanced solubility.

**Figure 7:**
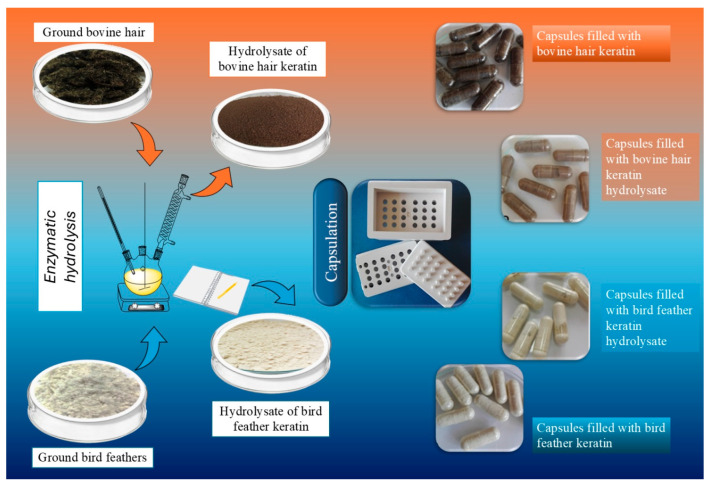
Process scheme showing the enzymatic hydrolysis of keratinous waste (bovine hair and poultry feathers), followed by drying, milling, and encapsulation of keratin hydrolysates or raw keratin powders in gelatin capsules for potential agricultural application.

Post-hydrolysis, the resulting solutions were dried at 30 °C to a constant weight and then finely milled using a Retsch MM 400 ball mill from Retsch GmbH (Haan, Germany) at 20 Hz for 30 s. This yielded uniform powders of keratin hydrolysates derived independently from both bovine and avian sources. The complete workflow of keratin processing, including raw materials, hydrolysates, and final encapsulated forms, is presented in Figure 7.

For encapsulation, transparent gelatin capsules (size 1) were filled manually using a capsule-filling device. Four keratin-based compositions were prepared: (i) ground bovine hair keratin, (ii) ground bird feather keratin, (iii) enzymatic hydrolysate of bovine hair, and (iv) enzymatic hydrolysate of bird feathers. Each capsule contained approximately 0.4 ± 0.05 g of active material. The gelatin matrix not only ensured ease of handling and dosage standardization but also functioned as a protective barrier enabling gradual release upon exposure to moisture.

The encapsulated biocomposites were subsequently evaluated for their physicochemical stability (including thermal and pH sensitivity), biodegradability in soil, and their potential to enhance plant growth. This system is proposed as a sustainable and biodegradable nutrient delivery platform for use in agrobiotechnology and circular agriculture.

### 4.3. Hydrogel Preparation

The hydrogel component was prepared through the controlled crosslinking of poly(vinyl alcohol) (PVA) with sodium tetraborate, forming a three-dimensional polymeric network suitable for integration into latex-based agrotextile composites. The synthesis was carried out in three stages.

In the first stage, a 4% (m/v) aqueous solution of PVA was prepared. A total of 7.5 g of PVA was added to 180 g of hot water in a three-necked flask equipped with a mechanical stirrer, reflux condenser, and thermometer. The mixture was stirred continuously at 80 °C until complete dissolution of the polymer. After cooling to room temperature, the solution was quantitatively transferred to a 200 mL volumetric flask and brought to volume with distilled water.

In the second stage, a 2% (m/v) sodium tetraborate solution was prepared by dissolving 18.37 g of tetraborate in 90 mL of hot water. After ensuring complete dissolution, the solution was cooled to room temperature, filtered, transferred to a 100 mL volumetric flask, and diluted to volume with distilled water.

To ensure reproducibility, five replicate stoichiometric titrations were carried out to determine the optimal crosslinking ratio between the PVA solution and the sodium tetraborate solution. The optimal solution was prepared using a diluted sodium tetraborate solution was prepared by mixing equal volumes of the stock solution and distilled water, and 40 mL of this solution was placed in a 50 mL beaker. A diluted PVA solution was prepared at a 9:1 weight ratio of stock solution to distilled water, and 50 mL was placed in a 100 mL beaker. Subsequently, 10 mL aliquots of the diluted PVA solution were transferred into 25 mL beakers and titrated dropwise with the diluted sodium tetraborate solution using a single-channel glass pipette until complete gelation occurred. In each of the five replicate titrations, the gel point was reached after the addition of 0.9 mL of the diluted sodium tetraborate solution. This value was taken as the effective crosslinking ratio. The optimal stoichiometric ratio was subsequently used to prepare latex–hydrogel mixtures for impregnation of cotton textile substrates. The final compositions of the latex–hydrogel coatings are presented in Table 3.

### 4.4. Cotton Matrix Coating with Latex–Hydrogel Compositions

Latex–hydrogel mixtures were prepared according to the formulations summarized in Table 1 and subsequently applied to cotton textile substrates to produce biodegradable agrotextiles. Rectangular sections of cotton fabric measuring 50 × 20 cm were cut and used as carriers for the coating process. Approximately 70 g of the latex–hydrogel mixture was evenly distributed over the surface of each textile using a standard paint roller, ensuring uniform application. The coated fabrics were left to dry under ambient conditions for 72 h. Following initial drying, thermal stabilization was carried out in a forced-air circulation oven from BINDER GmbH (Tuttlingen, Germany) at 120 °C for 5 min to enhance structural integrity and adhesion of the coating.

In an additional series of samples, gelatin was incorporated into the latex–hydrogel matrix to enhance the biodegradability and nutrient delivery potential of the final agrotextile. For this purpose, a 3% (w/v) aqueous solution of gelatin was prepared and added to the latex–hydrogel mixture at a ratio of 2.25 parts by weight relative to the total composite mass. After thorough dispersion of the gelatin into the mixture, the resulting formulation was applied to cotton textiles following the same procedure as described above. The coated fabrics were dried at room temperature for 72 h and subsequently thermally stabilized at 120 °C for 5 min.

## 5. Methods of Analysis

### 5.1. Biological Degredation

Latex–hydrogel coated textile samples in paddle-shaped form were placed in labeled containers filled with universal potting soil (pH 6.0–7.0). The containers were transferred to a climate chamber (MEMMERT HPP 108, Memmert GmbH + Co. KG, Schwabach, Germany) set at 30 °C and 80% relative humidity. The degradation process lasted 30 days.

Gelatin capsules—both empty and filled with keratin hydrolysates—were placed in small containers containing soil with a pH of 5.5–6.0. Each capsule was covered with a thin layer of soil, which was gently moistened with water. At 6, 24, and 48-h intervals, the soil was uncovered to inspect the degree of capsule decomposition. All procedures were conducted under standard laboratory conditions.

In order to compare the retention of mechanical properties after soil exposure, an empirical aging coefficient was calculated according to Equation (1):

(1) $$S=\frac{{T_{S}}^{\prime }\cdot {E_{b}}^{\prime }}{T_{S}\cdot E_{b}}$$

where: *T_S_*—tensile strength before aging (MPa); *E_b_*—elongation at break before aging (%); *T_S_*′—tensile strength after aging (MPa); *E_b_*′—elongation at break after aging (%)

### 5.2. Thermo-Oxidative Aging

Thermo-oxidative aging of the latex–hydrogel-coated textile samples was carried out to assess the thermal durability and structural stability of the composites under accelerated aging conditions. Paddle-shaped specimens were placed in forced-air circulation oven from BINDER GmbH (Tuttlingen, Germany) and exposed to a constant temperature of 70 °C for 7 days. After the aging period, the samples were removed and used for further evaluation of mechanical properties and color changes.

### 5.3. Tensile Strength and Elongation at Break

The mechanical performance of the prepared agrotextile samples was evaluated by tensile testing. Measurements were carried out using a Zwick 1422 universal testing machine from ZwickRoell GmbH & Co. KG (Ulm, Germany). Both non-aged samples and samples subjected to thermo-oxidative and biological aging were tested in order to assess the effect of environmental exposure on mechanical stability.

The specimens were prepared in the form of dumb-bell test pieces with a narrow-section width of 4 mm and total length of 70 mm. Their thickness was measured using a digital thickness gauge (Digital Thickness Gage, LIMIT 0–15 mm, Luna AB, Alingsås, Sweden), and the measured cross-section was used to calculate tensile strength. The specimens were mounted in the machine grips and subjected to uniaxial tensile loading with an initial preload of 0.1 N and a crosshead speed of 500 mm/min until break. Tensile strength (TS) was expressed in MPa, and elongation at break (Eb) was expressed as percentage of the gauge length.

### 5.4. Shore’s Scale Hardness

The surface hardness of the latex–hydrogel coated agrotextile samples was determined using a Shore type 00 durometer from ZwickRoell GmbH & Co. KG (Ulm, Germany). This method is specifically suited for soft polymeric and elastomeric materials. The instrument applied a contact force of 111.1 cN, and the measurement range spanned from 0 to 100 Shore degrees (°Sh).

Hardness was evaluated by quantifying the resistance of the sample to indentation using a 3/32-inch diameter spherical indenter (ZwickRoell GmbH & Co. KG Ulm, Germany). The test involved recording the depth of penetration under constant force, where lower penetration depths indicated higher material hardness. This parameter provides an indirect measure of the composite’s surface integrity and degree of crosslinking, which are critical for predicting mechanical durability under field conditions.

### 5.5. Fourier-Transformed Infrared Spectroscopy

Fourier Transform Infrared (FTIR) spectroscopy was employed to analyze the chemical structure of the hydrogel-coated textile composites using a Nicolet 6700 spectrometer (Thermo Fisher Scientific, Waltham, MA, USA). Spectral data were collected for non-aged samples as well as those subjected to thermo-oxidative and soil aging processes.

The spectra were recorded in the mid-infrared region, ranging from 4000 to 400 cm^−1^, capturing characteristic absorption bands associated with functional groups present in the polymer matrix. The analysis was performed in transmission mode, and the resulting spectra—expressed as transmittance versus wavenumber—enabled the identification of molecular changes and degradation patterns induced by environmental aging. FTIR analysis was instrumental in confirming the structural integrity and chemical modifications within the composites over time.

### 5.6. Colour Change Analysis

The evaluation of colour change in the agrotextile samples was conducted using a CM–3600d spectrophotometer (Konica Minolta Sensing Europe B.V., Warrington, UK), employing the CIELab colour space system. This method quantifies colour perception based on three parameters: L* (lightness), a* (red-green), and b* (yellow-blue), enabling precise assessment of visual changes induced by environmental factors. Samples subjected to thermo-oxidative aging and biological degradation were analyzed and compared against non-degraded reference samples to determine the extent of colour alteration.

### 5.7. Equilibrium Swelling

The equilibrium swelling behavior of the latex–hydrogel composites was evaluated by total immersion in distilled water. Test specimens, with an initial dry mass of approximately 30–40 mg, were weighed and then immersed in distilled water at ambient temperature for 48 h to reach equilibrium swelling. After removal from the liquid, excess surface water was gently removed and the swollen mass was measured immediately.

The equilibrium swelling ratio was calculated according to Equation (2):

(2) $$Q_{w}=\frac{m_{s}-m_{d}}{m_{d}}$$

where $m_{s}$ is the swollen mass after immersion and $m_{d}$ is the initial weight of the sample.

To compare the compactness of the polymer network, a relative crosslinking coefficient was calculated as the reciprocal of the equilibrium swelling ratio, according to Equation (3):

(3) $$\alpha_{c}=\frac{1}{Q_{w}}$$

where $\alpha_{c}$ is an empirical parameter used for comparative evaluation of the tested formulations. Higher $\alpha_{c}$ values correspond to lower swelling and thus to a more compact network structure.

### 5.8. Solubility in Water

The solubility of gelatin capsules was assessed under standard laboratory conditions using water as the solvent medium. Both empty capsules and those filled with biologically active keratin-based material were tested. Each capsule was placed in an individual vessel containing a fixed volume of water, and the dissolution process was monitored. Water solubility was evaluated qualitatively by visual observation of capsule swelling, rupture time, dispersion appearance, and sedimentation behavior.

### 5.9. pH Measurement After Capsule Dissolution

The pH of water following the dissolution of gelatin capsules was measured using a calibrated pH meter (pH-Meter Basic 20+, Crison Instruments, S.A., Alella, Spain). Both empty capsules and those filled with biocomponents were dissolved in distilled water prior to analysis. All measurements were performed under standard laboratory conditions.

### 5.10. Thermal Stability of Gelatin Capsules

The thermal stability of gelatin capsules was evaluated by exposing them to defined temperature conditions. Capsules were placed in a thermal chamber at temperatures of 30 °C and 50 °C, as well as under refrigeration at −4 °C and freezing at −20 °C. Observations were recorded daily over a five-day period to monitor physical changes in capsule integrity.

### 5.11. Plant Growth Assessment

Latex–hydrogel mats were placed in soil with a pH of 5.5–6.0, and dwarf bean seeds (*Phaseolus vulgaris*) were sown directly onto the mats. A parallel test was conducted using 5 gelatin capsules filled with keratin-based biofertilizer, which were also embedded in the soil prior to sowing. Plant growth was monitored over a 14-day period.

The most effective material combinations, determined based on observed plant development, were selected for the fabrication of the final product—an agrotextile integrated with gelatin capsules containing bioactive fertilizer. In the final configuration, the capsules were affixed to the latex–hydrogel mats, and a new sowing of *Phaseolus vulgaris* was conducted on the modified material. This setup was used to assess the combined effect of the hydrogel matrix and encapsulated biocomponents on plant growth enhancement.

### 5.12. Statistical Analysis

All quantitative measurements were performed in five technical replicates for each tested material formulation (n = 5). The obtained data were analyzed using descriptive statistical analysis, and the results are presented as the arithmetic mean ± standard error of the mean (SEM).

The arithmetic mean was calculated according to Equation (4):

(4) $$\begin{array}{cccc} & \overset{\text{-}}{x}=\frac{1}{n}\sum_{i=1}^{n}x_{i} & & \end{array}$$

where $\overset{\text{-}}{x}$ is the mean value, $x_{i}$ is the individual measurement, and n is the number of independent replicates.

The standard deviation (*SD*) was calculated using Equation (5):

(5) $$\begin{array}{cccc} & SD=\sqrt{\frac{\sum_{i=1}^{n}(x_{i}-\overset{\text{-}}{x}{)}^{2}}{n-1}} & & \end{array}$$

where *SD* describes the dispersion of the measured values around the arithmetic mean.

The standard error of the mean (SEM) was then calculated according to Equation (6):

(6) $$\begin{array}{cccc} & SEM=\frac{SD}{\sqrt{n}} & & \end{array}$$

The *SEM* was used to estimate the uncertainty of the mean value obtained from the five independent replicate measurements.

## 6. Conclusions

This study showed that cotton-based agrotextiles can be modified with latex–hydrogel coatings and keratin-based capsule systems to obtain biodegradable materials with useful agricultural properties. Their behavior depended on the composition of the coating, especially the hydrogel content and the presence of gelatin, which together influenced water uptake, mechanical performance, and degradation. The results also indicated that keratin hydrolysates were more suitable than native keratin for capsule filling because they dissolved more readily and were therefore more likely to support early nutrient availability. Since the keratin was obtained from agro-industrial waste, the developed system also reflects a circular approach in which low-value residues are converted into functional agricultural materials. In this way, the study points to a practical route for designing sustainable agrotextiles whose properties can be adjusted according to the intended application.

## Figures and Tables

**Table 1:** Results for equilibrium swelling ratio and relative crosslinking coefficient.

	L	LH10	LH20	LH30	LH40	LH10Z	LH20Z	LH30Z	LH40Z
**Q_w_**	0.205 ± 0.007	0.481 ± 0.020	0.491 ± 0.019	0.432 ± 0.014	0.413 ± 0.018	0.273 ± 0.014	0.295 ± 0.009	0.425 ± 0.019	0.419 ± 0.020
**α** **_c_**	4.876 ± 0.210	2.078 ± 0.091	2.035 ± 0.080	2.312 ± 0.120	2.420 ± 0.110	3.660 ± 0.140	3.395 ± 0.140	2.351 ± 0.100	2.387 ± 0.130

**Table 2:** Results of the pH analysis after capsule dissolution.

Sample	pH
Water	7.38 ± 0.03
Gelatin Capsule	7.51 ± 0.04
Gelatin Capsule with Feather Keratin	7.03 ± 0.04
Gelatin Capsule with Feather Keratin Hydrolysate	8.02 ± 0.05
Gelatin Capsule with Bovine Hair Keratin	7.21 ± 0.04
Gelatin Capsule with Bovine Hair Keratin Hydrolysate	8.04 ± 0.05

**Table 3:** Composition of films applied on cotton matrix.

	Composition Name								
Substrates [*w*/*w* %]	L	LH10	LH20	LH30	LH40	LH10Z	LH20Z	LH30Z	LH40Z
LBSK latex	100	90	80	70	60	90	80	70	60
Hydrogel	0	10	20	30	40	10	20	30	40
Gelatin	0	0	0	0	0	2.25	2.25	2.25	2.25

## Data Availability

No data were used for the research described in the article.

## Supplementary Materials

The following supporting information can be downloaded at: https://www.mdpi.com/article/10.3390/molecules31091544/s1, Table S1: Comparative summary of formulation composition and selected physicochemical and aging properties of the latex–hydrogel agrotextiles.
