# Supplementary material for: Keratin-Integrated Latex–Hydrogel Coatings: Biopolymer Design for Functional Agrotextile Materials
Source: Molecules. 2026 May 6;31(9):1544. doi: 10.3390/molecules31091544 (PMC13165177; doi:10.3390/molecules31091544)
Supplement: Supplementary file 1 [file molecules-31-01544-s001.zip › molecules-4188028-supplementary.pdf]

Table S1. Comparative summary of formulation composition and selected physicochemical and aging properties of the latex–hydrogel agrotextiles.

Formulation	Latex (wt%)	Hydrogel (wt%)	Gelatin (wt%)	Biological aging coefficient	Thermo-oxidative aging coefficient	Tensile strength (MPa)	Surface hardness (Sh)	Equilibrium swelling ratio	Degree of crosslinking
**L**	100	0	0	0.80 ± 0.04	1.40 ± 0.07	9.8 ± 0.5	14.5 ± 0.6	0.205 ± 0.007	4.876 ± 0.210
**LH10**	90	10	0	0.23 ± 0.01	2.24 ± 0.11	10.5 ± 0.7	6.8 ± 0.5	0.481 ± 0.020	2.078 ± 0.091
**LH20**	80	20	0	2.19 ± 0.11	2.49 ± 0.12	11.0 ± 0.7	7.0 ± 0.6	0.491 ± 0.019	2.035 ± 0.080
**LH30**	70	30	0	1.62 ± 0.08	2.16 ± 0.11	14.7 ± 1.0	21.2 ± 0.8	0.432 ± 0.014	2.312 ± 0.120
**LH40**	60	40	0	1.80 ± 0.09	1.48 ± 0.07	14.2 ± 0.9	21.6 ± 0.7	0.413 ± 0.018	2.420 ± 0.110
**LH10Z**	90	10	2.25	0.25 ± 0.01	0.84 ± 0.04	11.8 ± 0.6	8.3 ± 0.6	0.273 ± 0.014	3.660 ± 0.140
**LH20Z**	80	20	2.25	0.56 ± 0.03	0.71 ± 0.04	11.6 ± 0.5	8.8 ± 0.6	0.295 ± 0.009	3.395 ± 0.140
**LH30Z**	70	30	2.25	0.72 ± 0.04	0.31 ± 0.02	13.4 ± 0.7	20.1 ± 0.5	0.425 ± 0.019	2.351 ± 0.100
**LH40Z**	60	40	2.25	1.90 ± 0.10	0.98 ± 0.05	14.1 ± 0.5	20.7 ± 0.6	0.419 ± 0.020	2.387 ± 0.130
